# Supplementary material for: Temporal dendritic heterogeneity incorporated with spiking neural networks for learning multi-timescale dynamics
Source: Nat Commun. 2024 Jan 4;15:277. doi: 10.1038/s41467-023-44614-z (PMC10766638; doi:10.1038/s41467-023-44614-z)
Supplement: Supplementary file 1 — Supplementary Information [file 41467_2023_44614_MOESM1_ESM.pdf]

# Supplementary Information for Temporal Dendritic Heterogeneity Incorporated with Spiking Neural Networks for Learning Multi-timescale Dynamics

**Hanle Zheng[1], Zhong Zheng[1], Rui Hu[1], Bo Xiao[1], Yujie Wu[2], Fangwen Yu[1], Xue Liu[1], Guoqi Li[3], Lei Deng[1,*]**

## Contents

**S1 Supplementary Tables** . . . . . **S1**

S1.1 Model configuration details for tasks used in ablation studies . . . . . S1

S1.2 Model configuration details for other tasks . . . . . S1

S1.3 Initialization of timing factors . . . . . S2

S1.4 Computational efficiency comparison between SNNs with dendritic heterogeneity and synaptic heterogeneity . . . . S2

S1.5 Computational efficiency comparison between DH-SNNs and LSTM . . . . . S2

S1.6 Accuracy comparison between DH-SNNs and prior methods for three-class EEG recognition . . . . . S2

**S2 Supplementary Figures** . . . . . **S3**

S2.1 Network structures . . . . . S3

S2.2 Influence of the membrane potential timing factors . . . . . S4

S2.3 Influence of the membrane potential reset mechanism . . . . . S5

S2.4 Two-dendritic-branch DH-SNNs without connection restriction for the multi-timescale spiking XOR problem . . . . S6

S2.5 Temporal characterization of SHD and SSC datasets . . . . . S7

S2.6 Influence of the number of dendritic branches . . . . . S8

S2.7 Influence of the parameter volume . . . . . S9

S2.8 Model robustness . . . . . S10

S2.9 Model generalization . . . . . S11

S2.10 Influence of the dendritic connection pattern . . . . . S12

S2.11 Details of implementation on neuromorphic hardware . . . . . S13

S2.12 Additional experiments for EEG-based emotion recognition . . . . . S14

S2.13 The NeuroVPR task for the robot . . . . . S15

**Supplementary References** . . . . . **S16**

[1]Center for Brain Inspired Computing Research (CBICR), Department of Precision Instrument, Tsinghua University, Beijing, China.
[2]Institute of Theoretical Computer Science, Graz University of Technology, Graz, Austria.
[3]Institute of Automation, Chinese Academy of Sciences, Beijing, China.
*Corresponding author. Email: leideng@mail.tsinghua.edu.cn

# S1 Supplementary Tables

## S1.1 Model configuration details for tasks used in ablation studies

**Table S1.** Model configuration details for tasks used in ablation studies.

| Task | Delayed spiking XOR problem | Multi-timescale spiking XOR problem | SHD | SSC |
|---|---|---|---|---|
| Input dimension | 20 | 40 | 700 | 700 |
| Output dimension | 2 | 2 | 20 | 35 |
| Number of neurons per layer | 16 | 16 | 64 | 200 |
| Seq. length | 150 | 100 | 1000 | 1000 |
| Learning rate | 1e-2 | 1e-2 | 1e-2 | 1e-2 |
| Loss | CE | CE | CE | CE |
| Batch size | 500 | 500 | 100 | 100 |
| Number of epochs | 100 | 100 | 100 | 100 |
| Learning rate decay | 0.1per50 | 0.1per50 | 0.5per20 | 0.1per25 |
| Learning rate decay type | Step | Step | Step | Step |

## S1.2 Model configuration details for other tasks

**Table S2.** Model configuration details for other tasks.

| Task | GSC | S-MNIST | PS-MNIST | TIMIT | DEAP | NeuroVPR |
|---|---|---|---|---|---|---|
| Input dimension | 120 | 1 | 1 | 39 | 32 | 2752 |
| Output dimension | 15 | 10 | 10 | 61 | 3 | 100 |
| Network structure | 120-r300-15<br>120-f200(3)-15 | 1-r64-r256-10 | 1-r64-r256-10 | 39-r256(bi)-61 | 32-f200-3<br>32-r100-3 | 2752-f512(2)-f256-100<br>2752-521-r512-100 |
| Seq. length | 101 | 784 | 784 | 1000 | 384 | 6 |
| Learning rate | 1e-2 | 1e-2 | 1e-2 | 1e-2 | 1e-2 | 1e-3 |
| Loss | CE | CE | CE | CE | CE | CE |
| Batch size | 200 | 128 | 128 | 64 | 200 | 20 |
| Number of Epochs | 150 | 150 | 150 | 150 | 200 | 100 |
| Learning rate decay | 0.5per25 | 0.1per50 | 0.1per50 | 0.1per60 | 0.1per100 | 0.5per25 |
| Learning rate decay type | Step | Step | Step | Step | Step | Step |

* The prefixes of "r" and "f" in network structure represent the layer of DH-SRNN and DH-SFNN, respectively.
* The "bi" represent the layer is a bidirectional layer. The number in the bracket represent the number of stacking layers with the same structure.

### S1.3 Initialization of timing factors

**Table S3. Initialization of timing factors.**

| Initialized distribution | Small | Medium | Large |
|---|---|---|---|
| $\hat{\beta}$ | $U(-4,0)$ | $U(0,4)$ | $U(2,6)$ |
| $\hat{\alpha}$ | $U(-4,0)$ | $U(0,4)$ | $U(2,6)$ |

### S1.4 Computational efficiency comparison between SNNs with dendritic heterogeneity and synaptic heterogeneity

**Table S4. Computational efficiency comparison between SNNs with dendritic heterogeneity and synaptic heterogeneity.**

| Model | #Parameters | #Computational operations for dendrite&synapse dynamics |
|---|---|---|
| 1-layer SFNN with dendritic heterogeneity | $MN+(2D+1)N$ | $2DN$ |
| 1-layer SFNN with synaptic heterogeneity | $3MN+N$ | $2MN$ |
| 1-layer SRNN with dendritic heterogeneity | $MN+N^2+(2D+1)N$ | $2DN$ |
| 1-layer SRNN with synaptic heterogeneity | $3MN+3N^2+N$ | $2MN+2N^2$ |

* We assume that a layer has $N$ neurons with $M$ inputs and $D$ represents the number of dendritic branches per neuron where the $D \ll M$.

### S1.5 Computational efficiency comparison between DH-SNNs and LSTM

**Table S5. Computational efficiency comparison between DH-SNNs and LSTM.**

| Dataset | Model | #Parameters | #MACs / timestep | Accuracy(%) | Efficiency Improvement (×) |
|---|---|---|---|---|---|
| SHD | LSTM[1] | 0.43M | 426.5K | 89.2% | 1× |
| | **DH-SRNN (1-layer, 2-branch)** | **0.05M** | **0.3K** | **91.34%** | **1456×** |
| SSC | LSTM[1] | 0.43M | 428.4K | 73.1% | 1× |
| | **DH-SRNN (1-layer, 8-branch)** | **0.19M** | **2.5K** | **79.64%** | **187×** |

* #MACs denotes the equivalent number of multiply-and-accumulate (MAC) operations. Each accumulation operation is regarded as 19.6% of a MAC operation according to the reported rule that an addition operation consumes $0.9pJ$ energy while a MAC operation consumes $4.6pJ$ energy by taking the 45nm CMOS technology as an example[2].
* The efficiency improvement is measured by the ratio of accuracy over #MACs / timestep.

### S1.6 Accuracy comparison between DH-SNNs and prior methods for three-class EEG recognition

**Table S6. Accuracy comparison between DH-SNNs and prior methods for three-class EEG recognition.**

| **Model** | **#Parameters** | **Accuracy** |
|---|---|---|
| MLP[3] | 0.04M | Valence: 49.5%<br>Arousal: 46.0% |
| CNN[4] | 0.12M | Valence: 66.8%<br>Arousal: 57.6% |
| SCNN[5] | 0.90M | Valence: 70.23%<br>Arousal: 70.25% |
| **DH-SFNN (1-layer, 8-branch)** | **0.01M** | **Valence: 77.46%**<br>**Arousal: 80.23%** |

## S2 Supplementary Figures

### S2.1 Network structures

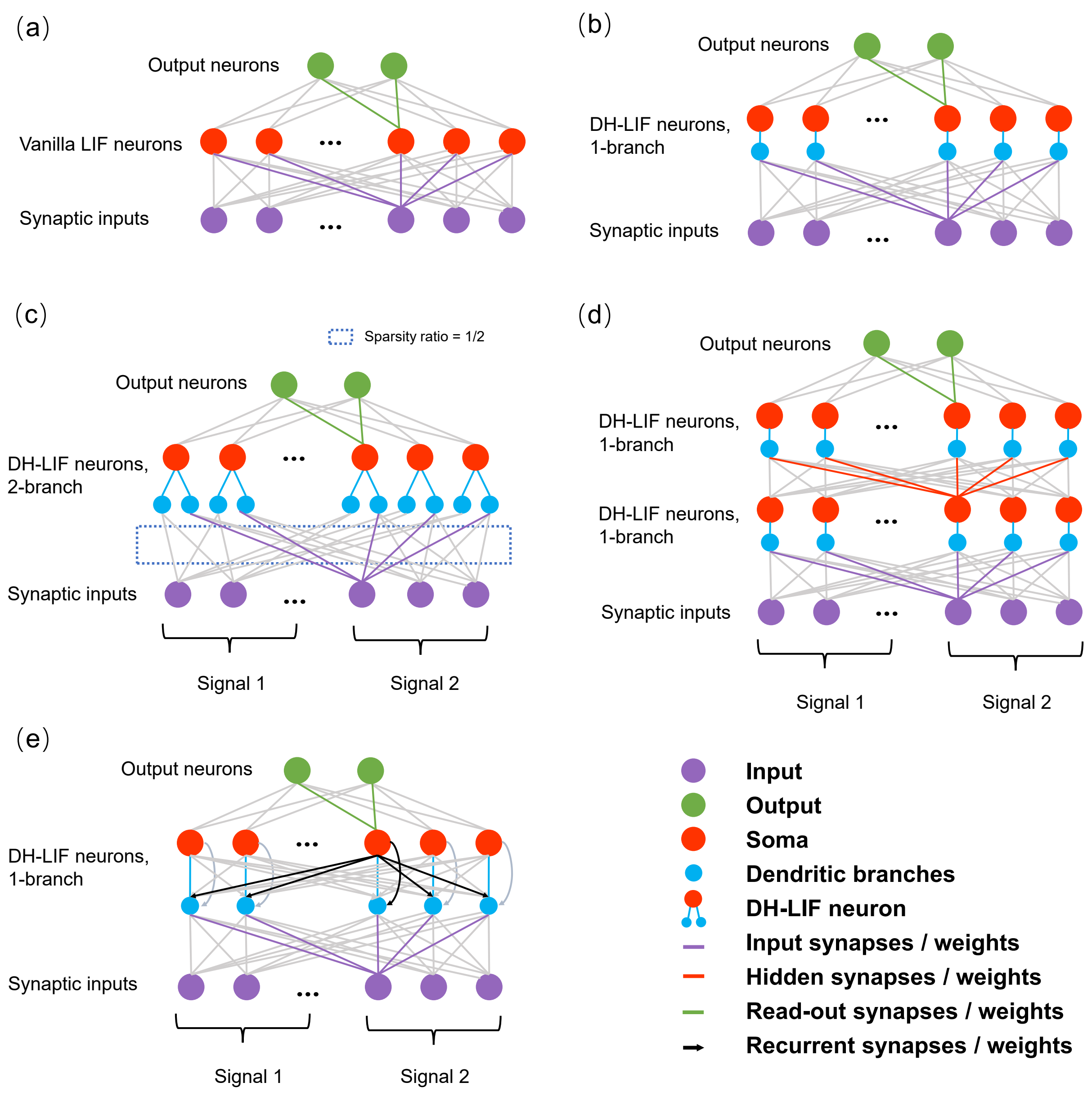


**Figure S1. Network structures.** **(a)** Vanilla SFNN and **(b)** DH-SFNN with one dendritic branch in each neuron used by the delayed and multi-timescale spiking XOR problems. **(c)** One-layer DH-SFNN with two dendritic branches in each neuron, **(d)** two-layer DH-SFNN with one dendritic branch in each neuron, and **(e)** one-layer DH-SRNN with one dendritic branch in each neuron used by the multi-timescale spiking XOR problem. The network structures used on SHD and SSC datasets are similar to above structures and the differences lie in the number of dendritic branches in each neuron, the number of neurons per layer, and the number of hidden layers, which are provided in Table S1.

### S2.2 Influence of the membrane potential timing factors

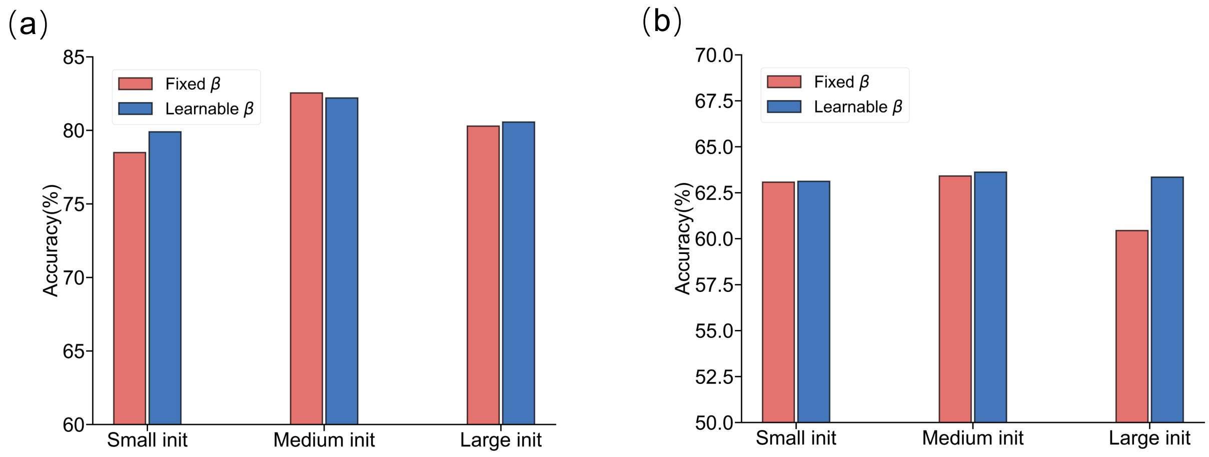


**Figure S2. Influence of the membrane potential timing factors.** Comparing recognition accuracy of DH-SFNNs with fixed or learnable membrane potential timing factors under different initialization on **(a)** SHD and **(b)** SSC datasets. The sampling time interval is $dt$=1$ms$. Each model only has one single layer and one dendritic branch in each neuron. The timing factors of dendritic branches are learnable and initialized with a large initialization. The results imply that learning membrane potential timing factors can slightly improve accuracy in most situations, while can boost accuracy when the initialized distribution is inappropriate. For example, the small initialization of membrane potential timing factors on SHD might lose the long-term memory of the dominant low-frequency components while the large initialization on SSC might lose the tracking capability of the rich high-frequency components, which can be alleviated by learning.

### S2.3 Influence of the membrane potential reset mechanism

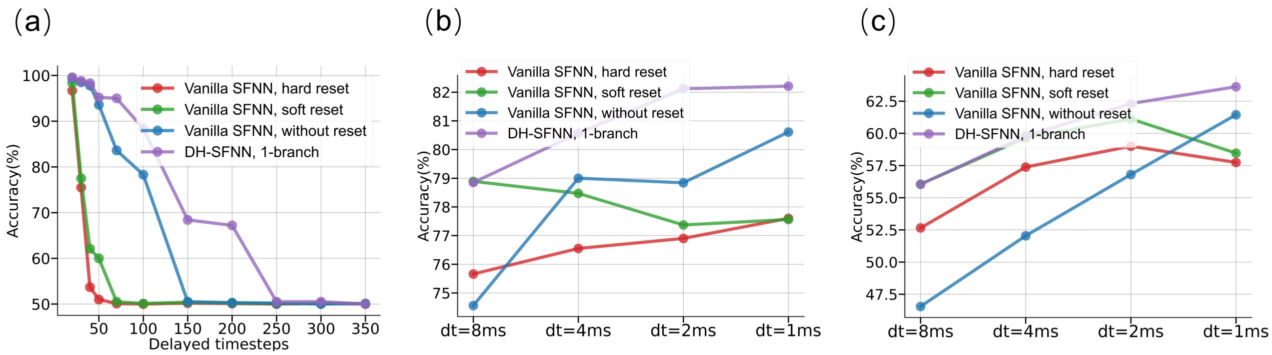


**Figure S3.** **Influence of the membrane potential reset mechanism.** Comparing recognition accuracy of vanilla SFNNs with different membrane potential reset mechanisms and DH-SFNNs with one dendritic branch in each neuron and one layer. The long-term memory is tested when performing tasks including **(a)** the delayed spiking XOR problem, **(b)** SHD and **(c)** SSC datasets. The timing factors of membrane potentials and dendritic currents are initialized with a medium distribution and a large distribution, respectively, both of which are learnable.

## S2.4 Two-dendritic-branch DH-SNNs without connection restriction for the multi-timescale spiking XOR problem

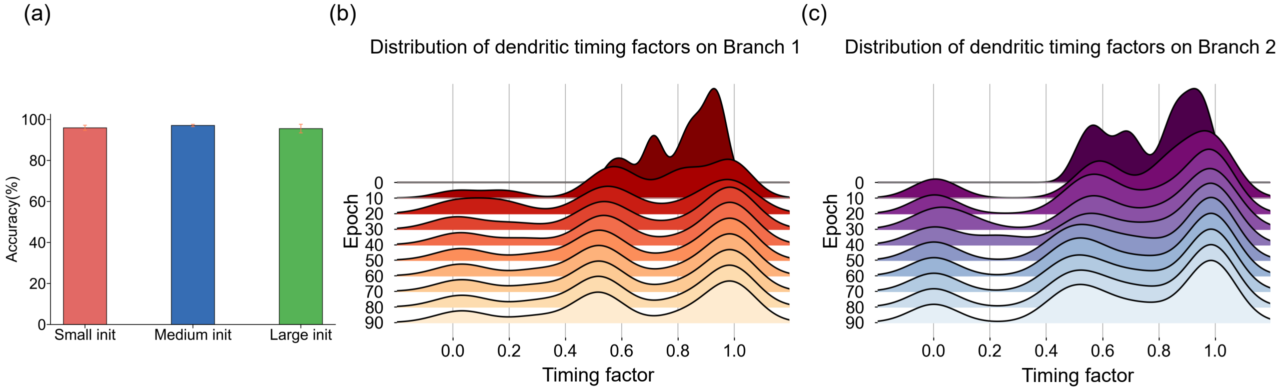


**Figure S4. Two-dendritic-branch DH-SNNs without connection restriction for the multi-timescale spiking XOR problem.** **(a)**, Comparing recognition accuracy of two-dendritic-branch DH-SFNNs under different initializations of dentritic timing factors. The evolving of $\boldsymbol{\alpha}$ on **(b)** Branch 1 and **(c)** Branch 2 under the medium initialization during training. In the experiments, Signal 1 and Signal 2 are randomly connected to the two dendritic branches without connection restriction. Two-dendritic-branch DH-SFNNs succeed in handling the problem. The KDE lines of $\boldsymbol{\alpha}$ distributions gradually exhibit three peaks different from the initialization, which evidences that the learning process can make the dendritic timing factors acquire selectivity to multiple timescales of input signals. In above experiments, unless otherwise specified, the timing factors of membrane potentials are initialized following a medium distribution and are learnable during training. KDE line, kernel density estimate line.

### S2.5 Temporal characterization of SHD and SSC datasets

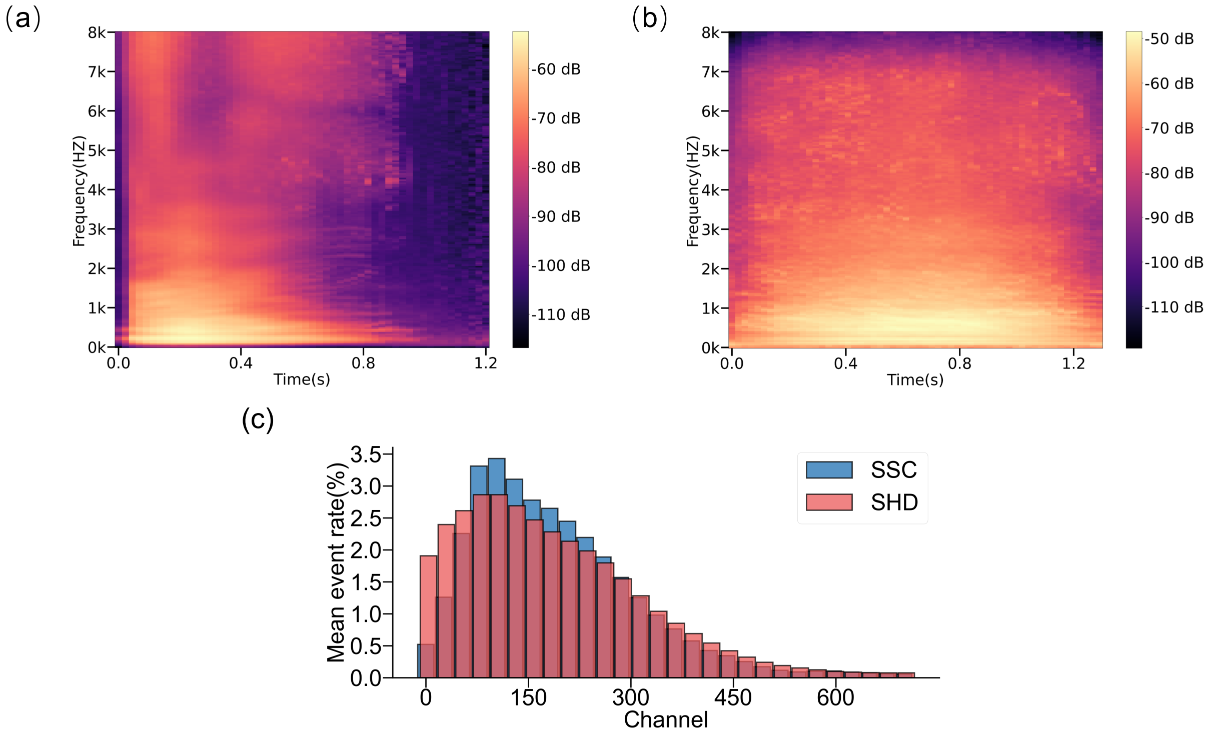


**Figure S5. Temporal characterization of SHD and SSC datasets.** The spectrograms of the original waves of **(a)** SHD and **(b)** SSC datasets. Here each sample is temporally divided into time bins, and the spectrogram is obtained by conducting Fast Fourier transform for all time bins. The final spectrogram of a dataset is obtained from averaging spectrograms of all samples. Apparently, the average spectrogram of SSC demonstrates richer temporal components at different frequency levels than that of SHD, which also reflects the higher complexity of SSC. **c,** Temporal heterogeneity of spike events from SHD and SSC datasets by showing the mean firing rate of the differenc channels.

### S2.6 Influence of the number of dendritic branches

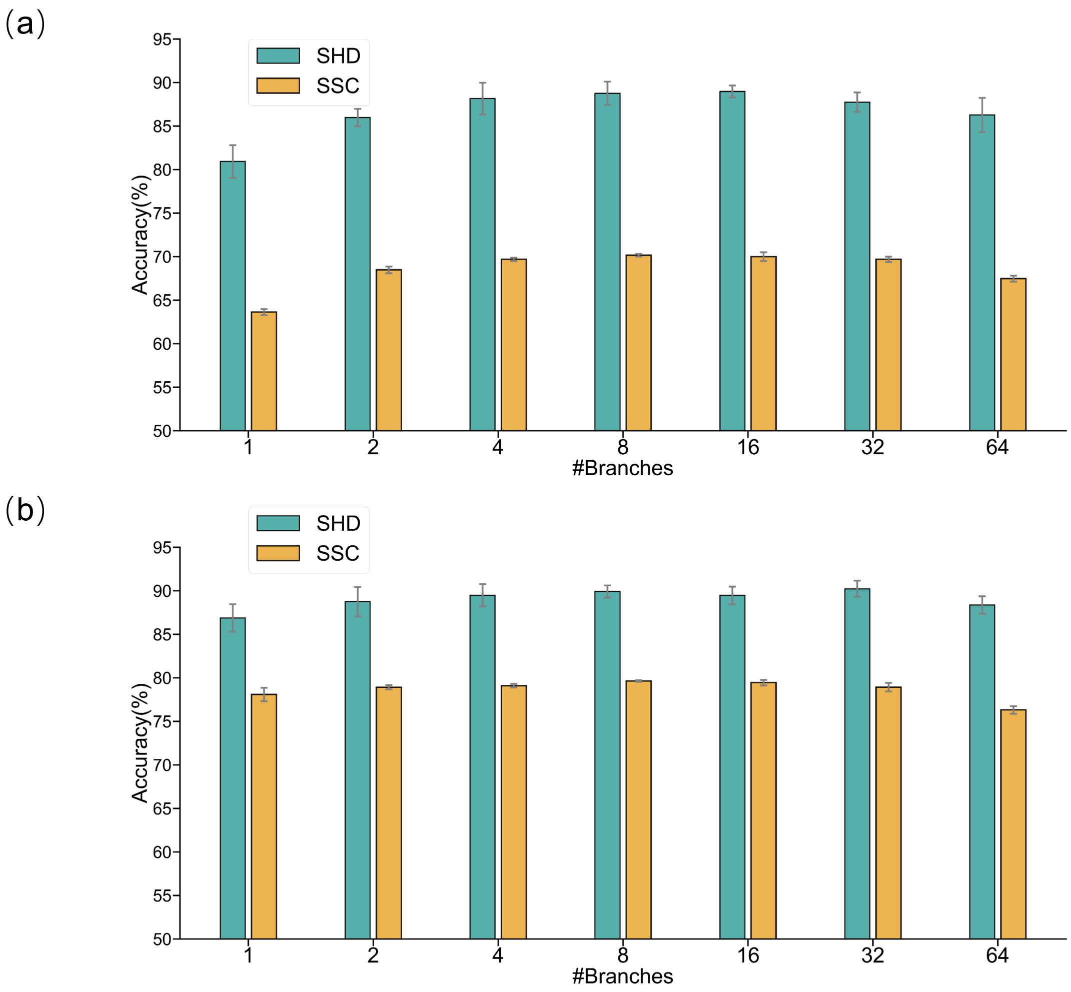


**Figure S6. Influence of the number of dendritic branches.** Comparing recognition accuracy of **(a)** DH-SFNNs and **(b)** DH-SRNNs with different number of dendritic branches on SHD and SSC datasets. The timing factors of membrane potentials and dendritic currents are initialized with a medium distribution and a large distribution, respectively, both of which are learnable. Each model only has one single layer. The accuracy can be improved as the number of dendritic branches properly grows, while can be saturated and even degraded when exceedingly grows. The standard deviations (presented as error bars) represent 5 repeated trials.

### S2.7 Influence of the parameter volume

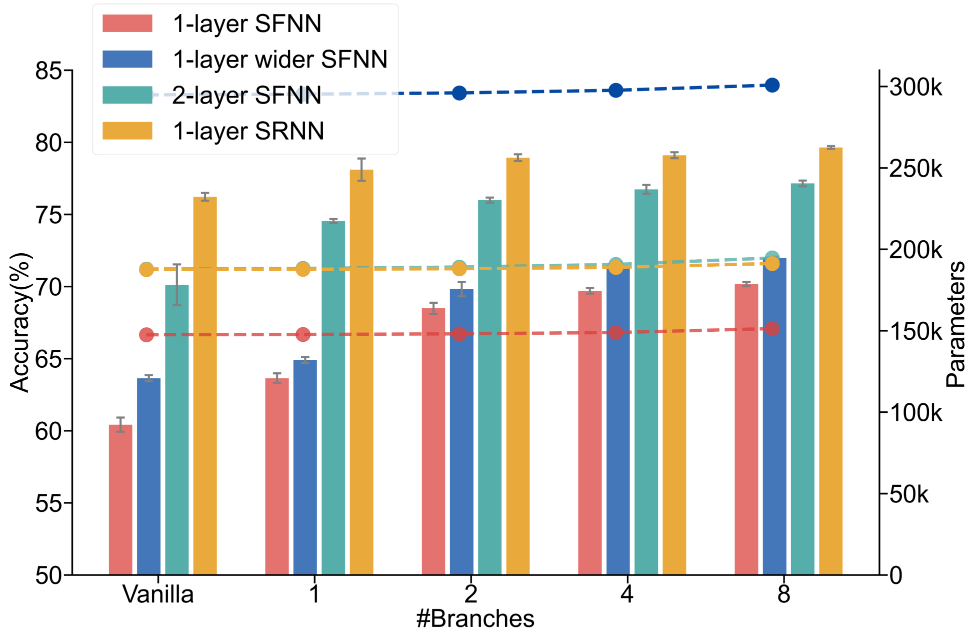


**Figure S7. Influence of the parameter volume.** Comparing recognition accuracy and the parameter volume of vanilla SNNs and DH-SNNs with different dendritic branches on the SSC dataset. The models include one-layer SFNNs, one-layer SFNNs with a wider structure which doubles the number of neurons per layer, two-layer SFNNs, and one-layer SRNNs. The bars present their recognition accuracy and the dotted lines demonstrate their parameter volume. Apparently, the one-layer SFNNs perform the worst with the minimum amount of parameters while the one-layer SFNNs with a wider structure perform the second worst even if they have the maximum number of parameters, which suggests that the performance improvement cannot be achieved by simply increasing the parameter volume. The standard deviations (presented as error bars) represent 5 repeated trials.

### S2.8 Model robustness

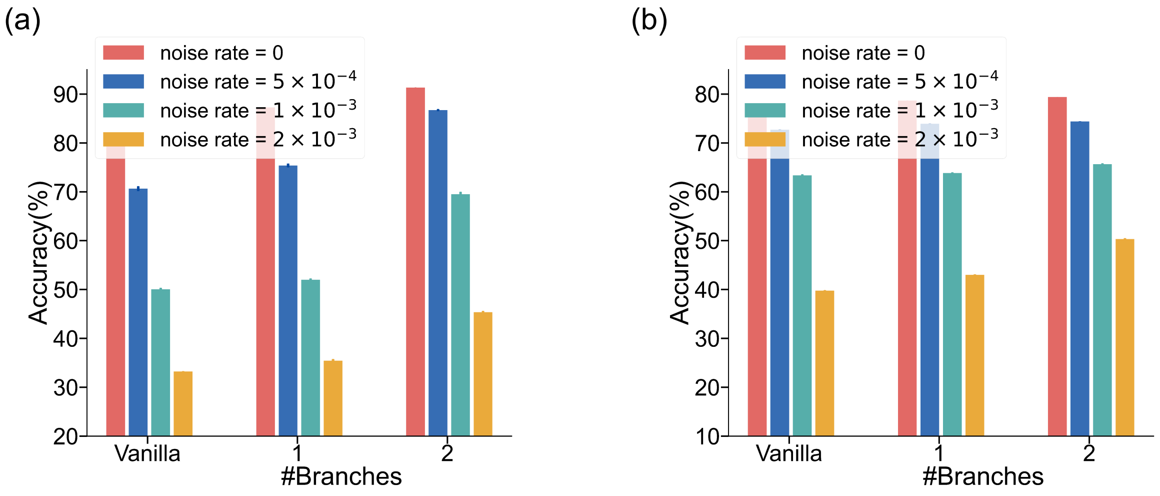


**Figure S8. Model robustness.** Comparing robustness of vanilla SRNNs and DH-SRNNs with different numbers of dendritic branches in resisting random spike noises on **(a)** SHD and **(b)** SSC datasets. The spike noises follow a Poisson distribution and have different noise rates. The testing models only have one single layer. The bars present recognition accuracy under different random noise rates. The results suggest that DH-SRNNs with multiple dendritic branches present better robustness against the interference of random spike noises.

### S2.9 Model generalization

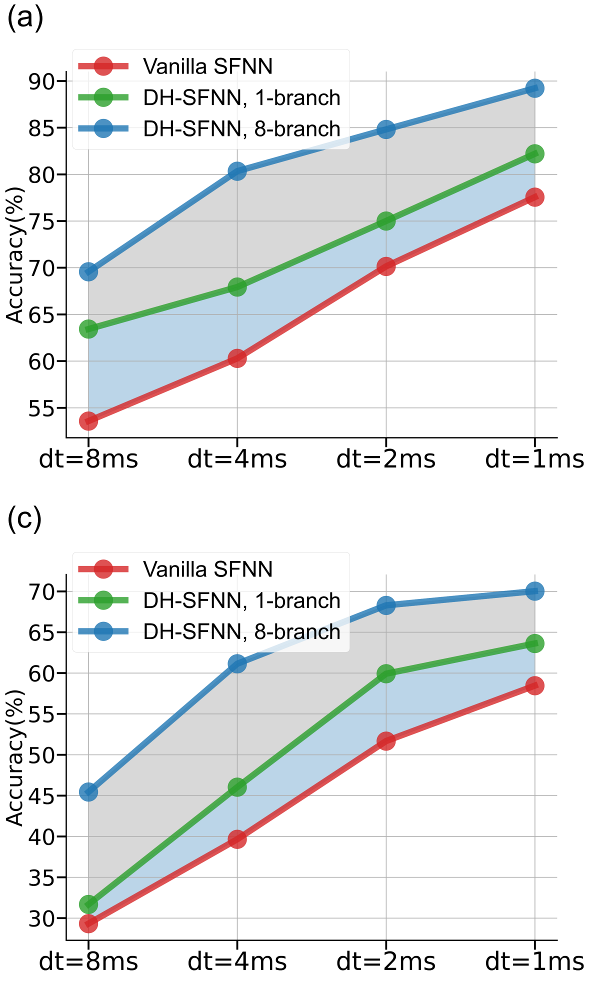


**Figure S9. Model generalization.** Comparing the generalization capability of **(a)** vanilla SFNNs and DH-SFNNs, **(b)** vanilla SRNNs and DH-SRNNs in adapting to different sampling time intervals on the SHD dataset. Comparing the generalization capability of of **(c)** vanilla SFNNs and DH-SFNNs, **(d)** vanilla SRNNs and DH-SRNNs in adapting to different sampling time intervals on the SSC dataset. All of testing models only have one single layer. DH-SFNNs and DH-SRNNs can have one or eight dendritic branches. The models are first pre-trained under the sampling time interval of $dt$=1$ms$ and then quickly fine-tuned under a new sampling time interval such as $dt$=2$ms$, $dt$=4$ms$, or $dt$=8$ms$. During fine-tuning, we fix massive synaptic weights and only train timing factors which just occupy a small fraction of total parameters. The fune-tuning process lasts only 20 epochs. At last, we evaluate the generalization capability by checking recognition accuracy under the new sampling time interval. The results imply that DH-SNNs with multiple dendritic branches can generalize to input information with variable timescales much better, owing to the natural temporal heterogeneity in the modeling of DH-SNNs.

## S2.10 Influence of the dendritic connection pattern

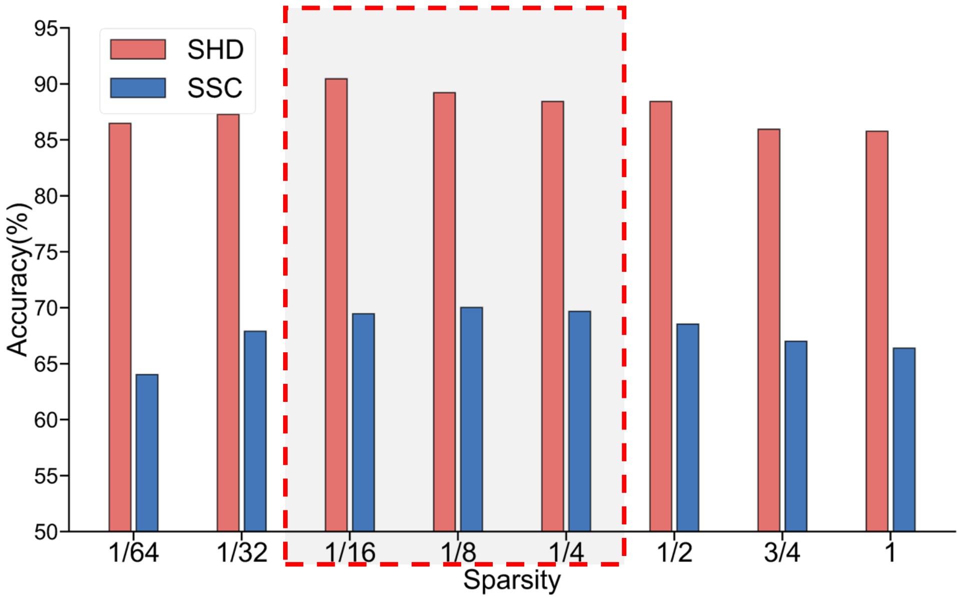


**Figure S10. Influence of the dendritic connection pattern.** Comparing recognition accuracy of one-layer DH-SFNNs with eight dendritic branches under different sparsity ratios on SHD and SSC datasets. The sparsity ratio represents the ratio of synaptic inputs connected to each dendritic branch over total synaptic inputs. The area inside the red dotted box shows the models that perform well in both tasks.

## S2.11 Details of implementation on neuromorphic hardware

(a)

| Operations | Input data type | Output data type |
|---|---|---|
| $\mathbf{V} = \mathbf{WX} + \mathbf{B}$ (FC) | $W$: INT8<br>$X$: Ternary | INT32 |
| $\mathbf{V} = \Sigma\mathbf{X} + \mathbf{B}$ (Sum) | INT8 | INT32 |
| **Memory data move** | INT8/Ternary | INT8/Ternary |
| **LIF dynamics** | INT32 | Spike: Ternary<br>Membrane potential: INT8 |
| **Data transmission** | INT8/Ternary | INT8/Ternary |

(b)

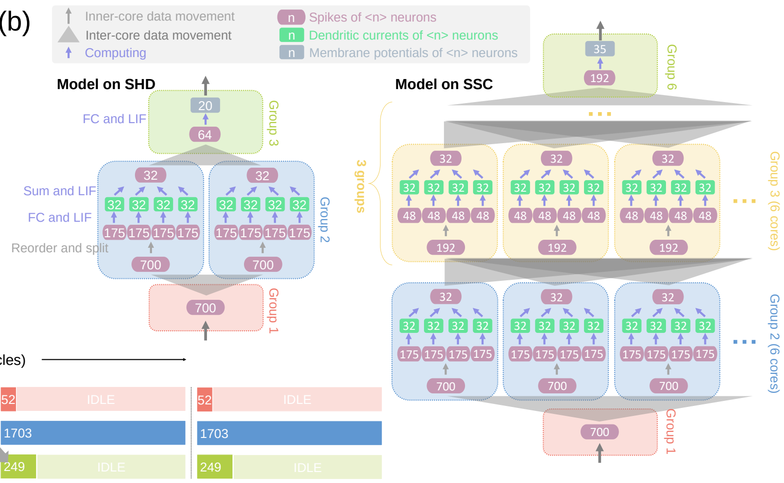


(c)

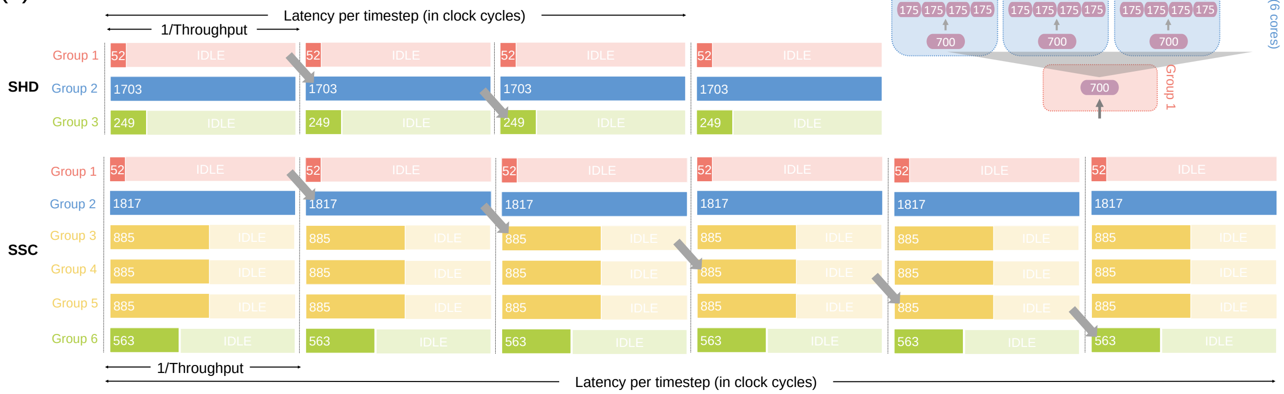


**Figure S11. Details of implementation on neuromorphic hardware. a,** Operations needed for implementing DH-SNNs on the TianjicX neuromorphic chip. **b,** Logical mapping of DH-SNNs on SHD and SSC datasets, wherein each box is an on-chip functional core. **c,** Chip execution timing schedule. The intra-core primitive instructions are executed in series for better utilization, the cores in each group run in parallel for shorter latency, and the groups are scheduled in a pipeline for higher throughput.

### S2.12 Additional experiments for EEG-based emotion recognition

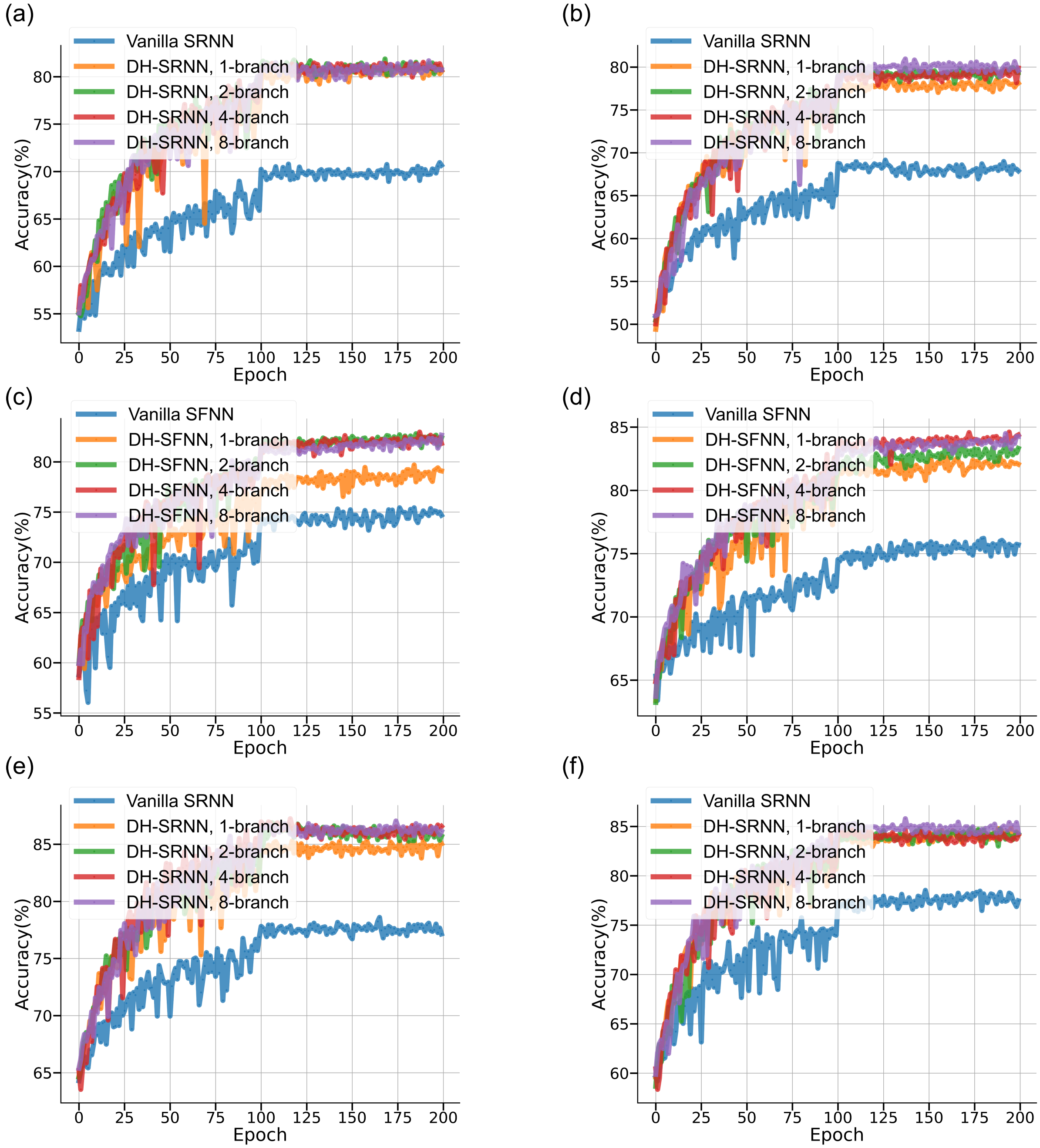


**Figure S12. Additional experiments for EEG-based emotion recognition.** Recognition accuracy curves of one-layer DH-SRNNs in three-class **(a)** valence and **(b)** arousal emotion recognition tasks. Recognition accuracy curves of one-layer DH-SFNNs in two-class **(c)** valence and **(d)** arousal emotion recognition tasks. Recognition accuracy curves of one-layer DH-SRNNs in two-class **(e)** valence and **(f)** arousal emotion recognition tasks. The dataset used for experiments is the DEAP dataset. The two-class version maps the levels of valence and arousal to two labels: low (score fewer than 5) and high (score higher than 5). Compared to the three-class emotion recognition, the accuracy results in the two-class emotion recognition are much higher. Compared to the results with DH-SFNNs, the accuracy gap presents a reduced trend between DH-SRNNs when varying the number of dendritic branches.

### S2.13 The NeuroVPR task for the robot

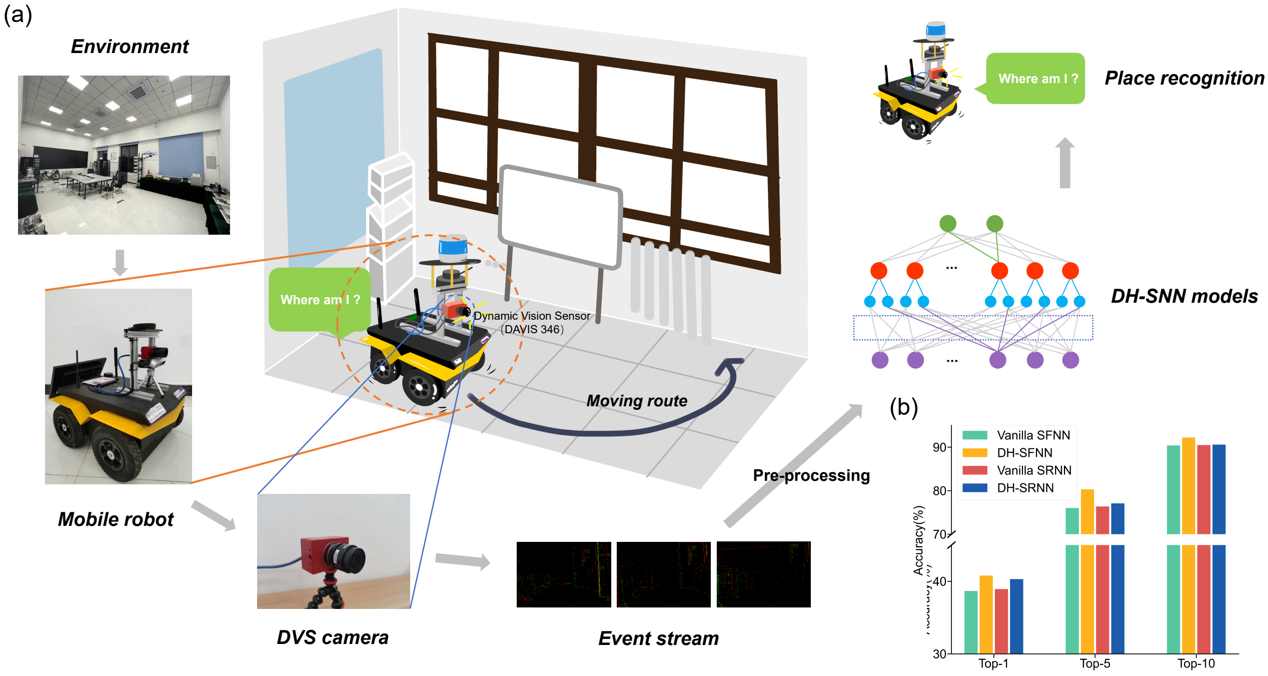


**Figure S13. The NeuroVPR task for the robot. a,** Illustration of the NeuroVPR task. The mobile robot recognizes the spatial position where it is through SNN models fed with a duration of the spike event steam generated by a DVS camera. **b,** Comparison of performance between vanilla SNNs and DH-SNNs with four dendritic branches in the NeuroVPR task. The results evidence the great potential of DH-SNNs in performing robotic tasks with rich temporal information.

## Supplementary References


**1.** Cramer, B., Stradmann, Y., Schemmel, J. & Zenke, F. The heidelberg spiking data sets for the systematic evaluation of spiking neural networks. *IEEE Transactions on Neural Networks Learn. Syst.* **33**, 2744–2757 (2022).

**2.** Horowitz, M. 1.1 computing's energy problem (and what we can do about it). In *2014 IEEE International Solid-State Circuits Conference Digest of Technical Papers (ISSCC)*, 10–14 (2014).

**3.** Jirayucharoensak, S., Pan-Ngum, S. & Israsena, P. Eeg-based emotion recognition using deep learning network with principal component based covariate shift adaptation. *The Sci. World J.* **2014**, 627892 (2014).

**4.** Tripathi, S., Acharya, S., Sharma, R., Mittal, S. & Bhattacharya, S. Using deep and convolutional neural networks for accurate emotion classification on deap dataset. *Proc. AAAI Conf. on Artif. Intell.* **31**, 4746–4752 (2017).

**5.** Islam, M. R. *et al.* Eeg channel correlation based model for emotion recognition. *Comput. Biol. Medicine* **136**, 104757 (2021).
